# DDIT4 Downregulation by siRNA Approach Increases the Activity of Proteins Regulating Fatty Acid Metabolism upon Aspirin Treatment in Human Breast Cancer Cells

**DOI:** 10.3390/cimb45060296

**Published:** 2023-05-28

**Authors:** Aistė Savukaitytė, Agnė Bartnykaitė, Justina Bekampytė, Rasa Ugenskienė, Elona Juozaitytė

**Affiliations:** 1 Oncology Research Laboratory, Institute of Oncology, Lithuanian University of Health Sciences, LT-50161 Kaunas, Lithuania; agne.bartnykaite@lsmuni.lt (A.B.); justina.bekampyte@lsmuni.lt (J.B.); rasa.ugenskiene@lsmuni.lt (R.U.); 2 Department of Genetics and Molecular Medicine, Lithuanian University of Health Sciences, LT-50161 Kaunas, Lithuania; 3 Institute of Oncology, Lithuanian University of Health Sciences, LT-50161 Kaunas, Lithuania; elona.juozaityte@lsmuni.lt

**Keywords:** aspirin, breast cancer, DDIT4, ACC1, CPT1A

## Abstract

Repositioning of aspirin for a more effective breast cancer (BC) treatment requires identification of predictive biomarkers. However, the molecular mechanism underlying the anticancer activity of aspirin remains fully undefined. Cancer cells enhance de novo fatty acid (FA) synthesis and FA oxidation to maintain a malignant phenotype, and the mechanistic target of rapamycin (mTORC1) is required for lipogenesis. We, therefore, aimed to test if the expression of mTORC1 suppressor DNA damage-inducible transcript (DDIT4) affects the activity of main enzymes in FA metabolism after aspirin treatment. MCF-7 and MDA-MB-468 human BC cell lines were transfected with siRNA to downregulate DDIT4. The expression of carnitine palmitoyltransferase 1 A (CPT1A) and serine 79-phosphorylated acetyl-CoA carboxylase 1 (ACC1) were analyzed by Western Blotting. Aspirin enhanced ACC1 phosphorylation by two-fold in MCF-7 cells and had no effect in MDA-MB-468 cells. Aspirin did not change the expression of CPT1A in either cell line. We have recently reported DDIT4 itself to be upregulated by aspirin. DDIT4 knockdown resulted in 1.5-fold decreased ACC1 phosphorylation (dephosphorylation activates the enzyme), 2-fold increased CPT1A expression in MCF-7 cells, and 2.8-fold reduced phosphorylation of ACC1 following aspirin exposure in MDA-MB-468 cells. Thus, DDIT4 downregulation raised the activity of main lipid metabolism enzymes upon aspirin exposure which is an undesired effect as FA synthesis and oxidation are linked to malignant phenotype. This finding may be clinically relevant as DDIT4 expression has been shown to vary in breast tumors. Our findings justify further, more extensive investigation of the role of DDIT4 in aspirin’s effect on fatty acid metabolism in BC cells.

## 1. Introduction

Aspirin, or acetylsalicylic acid, has been used to treat pain, fever, and inflammation and prevent heart attacks, strokes, and pathological clot formation [1]. A considerable body of data demonstrates that it also exerts antitumor action in various cancer types, including breast cancer (BC) [2,3,4,5,6,7,8,9,10,11,12,13,14,15,16], via a not fully clear mechanism [17]. BC is the most frequent cancer worldwide [18] and the leading cause of cancer death [19]. Thus, management of patients with BC requires new solutions to improve patients’ outcomes [20]. Repurposing aspirin for a more successful cancer treatment needs identification of predictive biomarkers to select patients who are most likely to benefit from the therapy [21].

Metabolic reprogramming is a well-established hallmark of cancer [22]. In addition to the well-known aerobic glycolysis (Warburg effect), cancer cells rewire their metabolism via additional strategies to sustain growth and survival [22]. It is increasingly recognized that cancer cells reprogram their lipid metabolism [23]. Tumor cells markedly elevate de novo fatty acid (FA) synthesis despite the availability of exogenous lipid sources to satisfy the demand for energy and macromolecules [24]. Increased expression of enzymes which regulate de novo FA synthesis have been associated with cancer risk and prognosis [25]. Elevated expression of sterol regulatory element-binding transcription factor 1 (SREBP1), the transcriptional activator of lipid biogenesis genes, has been linked to poor prognosis in BC [26]. Cancer cells are also known to dysregulate FA oxidation [27]. Mitochondrial FA oxidation produces adenosine triphosphate (ATP) to fuel cancer cells [28]. Genes involved in FA oxidation encourage cell proliferation and survival [25]. In vitro experiments have shown that the inhibition of FA oxidation suppresses the metastatic properties of BC cells [29]. Thus, targeting lipid metabolism may be an attractive therapeutic option, especially in BC, where it plays a central role in tumor biology [30].

Acetyl-CoA carboxylases (ACC) catalyze the ATP-dependent conversion of acetyl-CoA into malonyl-CoA [31]. Cytosolic ACC1 isoform is thought to be the critical enzyme in FA synthesis, since its enzymatic product malonyl-CoA serves as substrate for the synthesis of FAs [28]. The activity of ACC1 is regulated transcriptionally and post-transcriptionally [32]. Phosphorylation at a major regulatory site Ser79 by AMP-activated protein kinase (AMPK) results in the inhibition of ACC1 activity through the blockage of homodimer formation [32]. Breast cancer gene 1 (BRCA1) can also increase ACC1 phosphorylation at Ser79 by associating with and thus preventing ACC1 dephosphorylation [33]. It has been demonstrated that ACC1 silencing induces apoptosis in BC cells [34]. ACC1 mRNA expression is decreased in triple-negative BC compared to receptor-positive BC in both tissue samples and cell lines [35].

The key enzyme in mitochondrial FA oxidation is carnitine palmitoyltransferase 1 (CPT1) [23]. CPT1A is the most prevailing isoform which accomplishes the rate-limiting step in FA oxidation [36]. While the inner mitochondrial membrane is impermeable to fatty acyl-CoA thioesters, CPT1 catalyzes the formation of long-chain acylcarnitines which are able to cross the inner mitochondrial membrane [37]. An integrated genomics approach based on the use of gene expression signatures of oncogenic pathway activity identified CPT1A as a driver of proliferation in luminal BCs [38]. Long-term clinical follow-up data suggested high CPT1A mRNA expression in tumors to promote distant BC metastasis [39]. CPT1A has been recently suggested as a biomarker for BC disease-monitoring [37,40].

Activation of transcriptional lipogenesis regulator SREBP requires mechanistic target of rapamycin (mTORC1) [41]. mTORC1 was also shown to be necessary for de novo lipogenesis activated by serine/threonine kinase (AKT) [41]. We have recently shown that DNA damage-inducible transcript 4 (DDIT4) expression determines the phosphorylation of mTORC1 target eukaryotic translation initiation factor 4E-binding protein 1 (4E-BP1) upon aspirin treatment [42]. Considering that de novo lipogenesis requires mTORC1 and mTORC1 activity (assessed by 4E-BP1 phosphorylation) is affected by intracellular DDIT4 level after aspirin treatment, we aimed to investigate whether DDIT4 knockdown impacts aspirin effect on the activity of main enzymes in lipid metabolism. We found that DDIT4 downregulation increased the activity of ACC1 and CPT1A in MCF-7 cells and activated ACC1 in the MDA-MB-468 BC cell line upon aspirin treatment.

## 2. Materials and Methods

### 2.1. Cell Lines, Culture Conditions, and Treatment

Human BC cell lines MCF-7 (luminal subtype) and MDA-MB-468 (triple-negative subtype) were purchased from CLS Cell Line Service (Eppelheim, Germany) and grown as monolayers in *Dulbecco’s Modified Eagle’s medium* (DMEM; Sigma-Aldrich, St. Louis, MO, USA) supplemented with 10% fetal bovine serum (Gibco, Gaithersburg, MD, USA) and 100 U/mL penicillin with 100 µg/mL streptomycin (Gibco, Gaithersburg, MD, USA) and 2 mM L-glutamine (Gibco, Gaithersburg, MD, USA) at 37 °C in a humidified environment with 5% CO_2_. Exponentially growing cells were plated into 25 cm^2^ flasks for 24 h and then treated with 2 mM of aspirin for 24 h. For the transfection experiments, the cells were reverse-transfected at the time of plating into flasks. We used 2 mM of aspirin since it is an achievable plasma salicylate concentration obtained from hydrolysis of aspirin [43].

### 2.2. Chemicals and Antibodies

Aspirin was purchased from Sigma-Aldrich (St. Louis, MO, USA) and 0.5 M stock solution was prepared in water (pH 7) and frozen at −20 °C in small quantities to prevent freeze–thaw cycles. Working solutions were prepared before each experiment.

Antibodies against DDIT4 (#ab191871) and CPT1A (#ab128568) were obtained from Abcam (Cambridge, UK). Anti-phospho-ACC1 Ser79 (#PA5-17725) antibody and anti-β-actin (#AM4302) were purchased from Invitrogen (Waltham, MA, USA). Horseradish peroxidase-conjugated anti-rabbit (#65-6120), anti-mouse (#61-6520) and alkaline phosphatase-conjugated anti-mouse (#WP20006) secondary antibodies were bought from Invitrogen (Waltham, MA, USA).

### 2.3. Western Blot Analysis

Cells were lysed in radioimmunoprecipitation assay (RIPA) buffer (Abcam, Cambridge, UK), supplemented with protease (Sigma-Aldrich, St. Louis, MO, USA) and phosphatase inhibitor cocktails (Sigma-Aldrich, St. Louis, MO, USA) for 20 min on ice. Cell lysate was centrifuged at 8000× *g* for 20 min, and the supernatants were further assayed or stored at −20 °C until use. Protein concentration was determined using Pierce^TM^ BCA Protein Assay Kit (Thermo Fisher Scientific, Waltham, MA, USA), according to the manufacturer’s protocol. Forty micrograms of proteins were loaded in each lane for electrophoresis in 12% or 4–12% gradient SDS-PAGE. The resolved proteins were electrophoretically transferred onto a PVDF (polyvinylidene fluoride) membrane using a semi-wet transfer unit Mini Blot module (Invitrogen, Waltham, MA, USA). Membranes were blocked in optimal blocking buffer for each antibody for 45 min and incubated overnight at 4 °C with primary antibody against DDIT4 (1:1000), phospho-ACC1 Ser79 (1:1000), CPT1A (1:1000), or β-actin (1:2000). After washing three times using TBST (Tris buffered saline with tween 20), the blots were further incubated with HRP (for probing DDIT4 and phospho-ACC1) or AP-conjugated secondary antibodies (for β-actin and CPT1A). The blots were washed again with TBST three times, and the proteins were detected using SuperSignal^TM^ West Atto Ultimate Sensitivity Substrate(Thermo Fisher Scientific, Waltham, MA, USA; for DDIT4 and phospho-ACC1) or CDP-Star^®^ chemiluminescent substrate (for β-actin and CPT1A) from WesternBreeze Chemiluminescent Kit (Invitrogen, Waltham, MA, USA). The chemiluminescent reaction was captured using the iBright™ CL750 Imaging System (Invitrogen, Waltham, MA, USA). Densitometry analysis was performed with the iBright™ Analysis Software (Invitrogen, Waltham, MA, USA) in the Thermo Fisher Connect Platform. Β-actin was used as a loading control.

### 2.4. Cell Transfection

Cells were reverse-transfected using Lipofectamine^TM^ RNAiMAX transfection reagent (Invitrogen, Waltham, MA, USA) and 8 nM Silencer Select siRNA as per manufacturer’s instructions. The following siRNAs were employed: siRNA targeting DDIT4 (#s29166; Ambion, Austin, TX, USA), non-targeting siRNA (#4390843; Invitrogen, Waltham, MA, USA), and positive control siRNA against GAPDH (#4390849; Invitrogen, Waltham, MA, USA). Target gene knockdown efficiency was first measured in time course experiments (24–72 h) to select the time point with efficient silencing for the drug treatment. Transfection conditions were optimized to induce at least 70% target mRNA knockdown while minimizing cytotoxicity.

Gene knockdown efficiency was evaluated by quantitative reverse transcription-polymerase chain reaction (qRT-PCR) on a Quanstudio 3 Real-Time PCR System (Applied Biosystems, Foster City, CA, USA). RNA was extracted with the PureLink^TM^ RNA Mini Kit (Invitrogen, Waltham, MA, USA) and quantified using a Qubit^®^ 3.0 fluorometer (Life Technologies, Carlsbad, CA, USA) and the Qubit^TM^ RNA HS Assay Kit (Invitrogen, Waltham, MA, USA) following the manufacturer’s recommendations. cDNA was synthesized using the High-Capacity RNA-to-cDNA kit (Applied Biosystems, Foster City, CA, USA), as per manufacturer’s instructions. PCR reaction was performed with Taqman^TM^ Gene Expression Assays (Hs99999905_m1 for GAPDH and Hs01111686_g1 for DDIT4 quantification) and Taqman^TM^ Universal Master Mix II with UNG from Applied Biosystems (Foster City, CA, USA), following the manufacturer’s instructions. Relative quantification of target gene expression was determined by the comparative Ct method with β-actin as internal control. The transfection efficiency was verified by Western Blotting analysis.

### 2.5. Comparison of Protein Fold Change in Transfected Cells

Fold change due to aspirin (ASA) treatment was compared in DDIT4 siRNA-transfected cells (siDDIT4) to control siRNA transfected cells (siCT) as follows:

$$\left(\frac{expression\;post\;ASA\;treatment\;in\;siDDIT4\;cells}{expression\;post\;control\;treatment\;in\;siDDIT4\;cells}\right)versus(\frac{expression\;post\;ASA\;treatment\;in\;siCT\;cells}{expression\;post\;control\;treatment\;in\;siCT\;cells})$$

### 2.6. Statistical Analysis

Results have been expressed as mean ± standard deviation (SD) from independent experiments carried out in triplicate. Statistical significance was tested via Student’s *t*-test using SPSS 27.0.1 software (SPSS Inc., Chicago, IL, USA). Results were considered statistically significant at *p* < 0.05.

## 3. Results

### 3.1. Aspirin Inhibits ACC1 Activity in MCF-7 Cells

**Figure 1:**
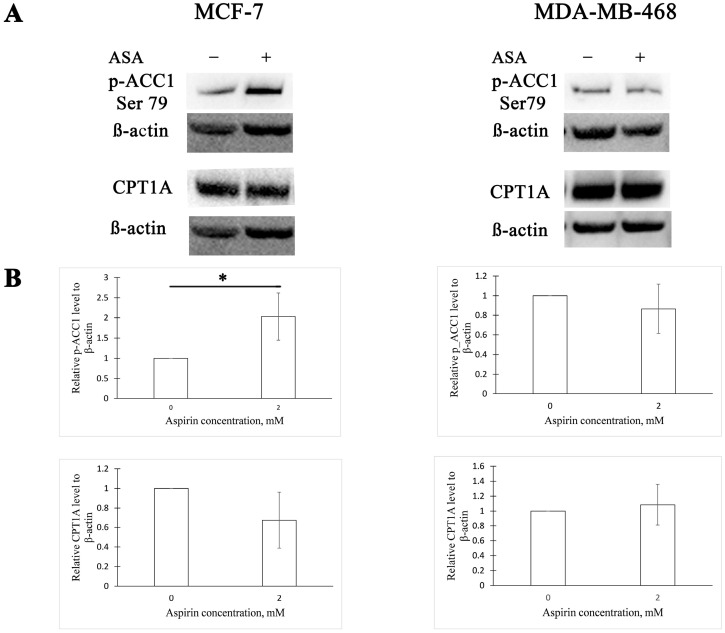
Aspirin increases acetyl-CoA carboxylase 1 (ACC1) phosphorylation in the MCF-7 cell line. (**A**) Representative Western blots of ACC1 phosphorylation and carnitine palmitoyltransferase 1A (CPT1A) expression in MCF-7 and MDA-MB-468 cells after 24 h aspirin (ASA) treatment. (**B**) Densitometric quantifications of p-ACC1 and CPT1A levels normalized to β-actin. The results are expressed as the mean ± SD (*n* = 3 in all graphs except CPT1A quantification in MCF-7 cells, where *n* = 4), * *p* value < 0.05.

We tested if aspirin affects the activity of ACC1 and expression of CPT1A in MCF-7 and MDA-MB-468 human BC cell lines. We observed that 24 h aspirin exposure increases ACC1 phosphorylation (at serine 79) by two-fold in the MCF-7 cell line and has no effect in MDA-MB-468 cells (Figure 1). Aspirin treatment for 24 h did not change the expression of CPT1A in either cell line (Figure 1).

### 3.2. DDIT4 Knockdown Enhances ACC1 Activity following Aspirin Treatment in BC Cell Lines

**Figure 2:**
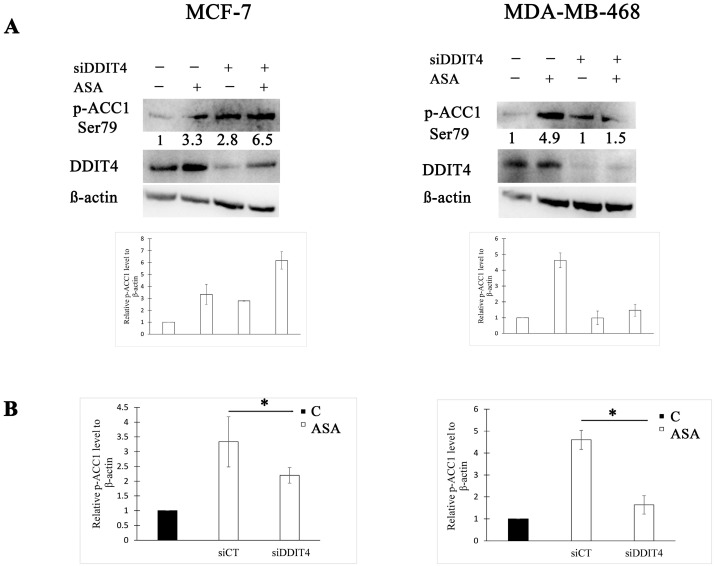
DNA damage-inducible transcript 4 (DDIT4) knockdown reduces ACC1 phosphorylation after aspirin treatment in breast cancer (BC) cell lines. (**A**) Representative Western blots and densitometric quantifications of p-ACC1 levels normalized to β-actin. MCF-7 and MDA-MB-468 cells were transfected with non-targeting (siCT) or DDIT4 siRNA (siDDIT4) for 24 h and treated with vehicle control (C) or 2 mM of aspirin (ASA) for the next 24 h before cell lysis. DDIT4 was probed on a different blot than p-ACC1. The numbers under the bands indicate densitometric quantifications of relative p-ACC1 level to β-actin. The results in the graphs are expressed as the mean ± SD (*n* = 3). (**B**) Comparison of p-ACC1 fold change after aspirin treatment between cells transfected with non-targeting siRNA and DDIT4 siRNA. The results are expressed as the mean ± SD (*n* = 3), * *p* value < 0.05.

We further asked whether DDIT4 expression determines ACC1 activity post-aspirin treatment in these BC cell lines. To manipulate DDIT4 expression, we adopted the RNA interference strategy. Compared to the control siRNA, DDIT4-targeting siRNA decreased ACC1 phosphorylation 1.5-fold in the MCF-7 cell line (Figure 2). In MDA-MB-468 cells, downregulation of DDIT4 caused a 2.8-fold reduction of the phosphorylated ACC1 amount following aspirin exposure (Figure 2). Thus, a lower DDIT4 level within these cell lines leads to a higher ACC1 activity after aspirin treatment as phosphorylation deactivates ACC1.

### 3.3. DDIT4 Knockdown Increases CPT1 Expression after Aspirin Treatment in MCF-7 Cells

**Figure 3:**
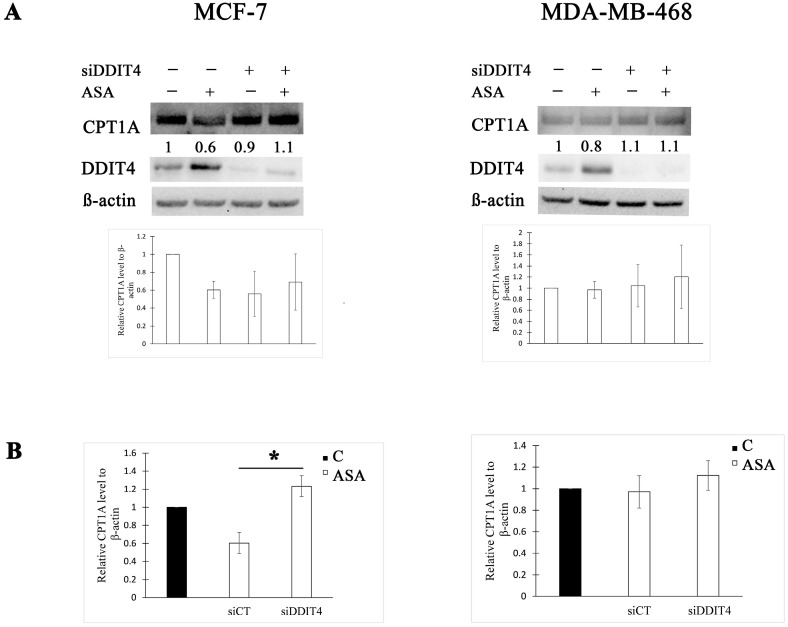
DDIT4 knockdown enhances CPT1A expression post aspirin treatment in MCF-7 cells. (**A**) Representative Western blots and densitometric quantifications of CPT1A levels normalized to β-actin. MCF-7 and MDA-MB-468 cells were transfected with non-targeting (siCT) or DDIT4 siRNA (siDDIT4) for 24 h and treated with vehicle control (C) or 2 mM of aspirin (ASA) for the next 24 h before cell lysis. DDIT4 was probed on a different blot than CPT1A in MCF-7 cell lysates. The numbers under the bands indicate densitometric quantifications of relative CPT1A level to β-actin. The results in the graphs are expressed as the mean ± SD (*n* = 3). (**B**) Comparison of CPT1A fold change after aspirin treatment between cells transfected with non-targeting siRNA and DDIT4 siRNA. The results are expressed as the mean ± SD (*n* = 3). * *p* value < 0.05.

Having established that DDIT4 downregulation using siRNA approach influences the effect of aspirin on phosphorylation of FA biosynthesis enzyme ACC1, we then examined whether it decides the impact on the rate-limiting enzyme in FA oxidation. We found that DDIT4 knockdown increased CPT1A expression by two-fold after treatment with aspirin in MCF-7 cells (Figure 3). DDIT4-targeting siRNA had no effect on CPT1A expression post aspirin treatment in MDA-MB-468 cells compared to control siRNA (Figure 3).

## 4. Discussion

Dysregulated lipid metabolism impacts multiple intracellular processes, such as membrane synthesis, energy metabolism, and cell signaling to support tumorigenesis and cancer progression [44]. Therefore, targeting abnormal lipid metabolism in cancer treatment is of substantial clinical interest [44].

The de novo pathway of FA synthesis plays an important role in mammary tumorigenesis, and ACC1 is the rate-limiting enzyme in this process [35]. Inhibition of lipogenic enzymes, including ACC1, has been recently reported as one of anticancer therapy strategies [45]. Aspirin has been demonstrated to reduce ACC activity through activation of its repressor AMPK in human hepatoma HepG2 cells, and the effect was ascribed to salicylic acid [46]. Aspirin-mediated inhibitory phosphorylation at serine 79 of ACC has also been shown in SUM159-PT human BC cell lines [47]. Downregulation of other critical enzymes in FA synthesis including SREBP1, stearoyl-CoA desaturase 1 (SCD1), fatty acid synthase (FASN), and ATP citrate lyase (ACLY) has been reported for aspirin in AU-565 and SKBR-3 human BC cells [48]. Quantification of fatty acid production through liquid chromatography–tandem mass spectrometry analysis also showed that aspirin attenuated the levels of lipid formation in BC cells [48]. Accordingly, suppression of FA synthesis via blockage of active state ACC1 and other lipogenesis enzymes may at least partly explain the antitumor properties of aspirin.

CPT1A is the rate-limiting enzyme in FA oxidation which is also implicated in malignant phenotype [35,38,39]. On the contrary to aspirin’s inhibitory effect on ACC1 and other FA synthesis enzymes, aspirin has been reported to promote the expression of FA oxidation genes CPT1 and MCAD at RNA and protein levels in human hepatoma HepG2 cell line [46]. The effect of aspirin on the increase in fatty acid oxidation has been also documented in mouse embryonic fibroblasts through analysis of ^3^H-labeled palmitate oxidation [49]. The authors suggested that the metabolic outcome of aspirin in vivo may depend on the expression of thioesterases that hydrolyze acetylsalicylic acid (aspirin) to salicylic acid since they found an opposite effect of salicylic acid on 3H-palmitate oxidation. Aspirin-mediated activation of fatty acid oxidation was further confirmed through the analysis of human urine samples collected after 7 days of aspirin treatment [50]. Promotion of FA oxidation upon aspirin treatment is consistent with the reported AMPK activation and resulting deactivation of ACC [46,47], because the catalytic product of ACC2 isoform malonyl-CoA is the natural inhibitor of CPT1A [28,51]. However, since FA oxidation plays a role in maintenance of the malignant phenotype, one may speculate that the induction of lipid oxidation may limit the effectiveness of aspirin in cancer treatment.

In this study, we first examined if aspirin influences the activity of ACC1 and CPT1A in MCF-7 and MDA-MB-468 human BC cell lines. We found an aspirin-mediated increase in ACC1 phosphorylation at serine 79 in the MCF-7 cell line, which is in line with previous reports [46,47]. However, aspirin exposure had no effect on ACC1 phosphorylation in MDA-MB-468 cells. Varying activities of the tested drug may be explained by different genetic backgrounds within these cells.

We did not detect alterations in CPT1A expression due to aspirin treatment in either of the tested cell lines. This is in contrast with a previous report where aspirin was noted to promote the expression of CPT1 in the human hepatoma cell line [46]. The different cancer types analyzed may be the cause of observed contrasting effects on CPT1. However, we cannot rule out the possibility that aspirin enhances FA oxidation in the tested BC cells via other mechanisms.

Next, we asked whether downregulation of DDIT4 expression alters the effect of aspirin on these enzymes. We chose to investigate the effect of DDIT4 expression based on a report by Porstmann et al. [41] which announced the necessity of mTORC1 for de novo lipid synthesis and on our recently published results uncovering the DDIT4 effect on mTORC1 activity upon aspirin treatment [42]. Our present data have shown for the first time that DDIT4 knockdown promotes the expression of CPT1A at the protein level and reduces phosphorylation of ACC1 (phosphorylation deactivates the enzyme) following aspirin treatment in MCF-7 BC cells and lowers the phosphorylation of ACC1 in MDA-MB-468 cells. Thus, DDIT4 downregulation leads to an undesired effect of aspirin on the activity of these lipid metabolism enzymes, as FA synthesis and oxidation are linked to malignant phenotype. These findings may be clinically relevant as DDIT4 expression has been shown to vary in breast tumors [52,53]. In this manner, the present findings are the basis for further, more extensive studies on the role of DDIT4 in aspirin’s effect on fatty acid metabolism.

## Data Availability

Data are contained within the article.
